# Design and Development of a Methodology Based on Expert Systems, Applied to the Treatment of Pressure Ulcers

**DOI:** 10.3390/diagnostics10090614

**Published:** 2020-08-20

**Authors:** Manuel Casal-Guisande, Alberto Comesaña-Campos, Jorge Cerqueiro-Pequeño, José-Benito Bouza-Rodríguez

**Affiliations:** Department of Design in Engineering, University of Vigo; 36208 Vigo, Spain; jcerquei@uvigo.es (J.C.-P.); jbouza@uvigo.es (J.-B.B.-R.)

**Keywords:** chronic wounds, pressure ulcers, expert systems, decision support systems, design science research

## Abstract

The medical treatment of chronic wounds, pressure ulcers in particular, burdens healthcare systems nowadays with high expenses that result mainly from their monitoring and assessment stages. Decision support systems applied within the ‘remote medicine’ framework may be of help, not only to the process of monitoring the evolution of chronic wounds under treatment, but also to facilitate the prevention and early detection of potential risk conditions in the affected patients. In this paper, the design and definition of a new decision-support methodology to be applied to the monitoring and assessment stages of the medical treatment process for pressure ulcers is proposed. Built upon the use and development of expert systems, the methodology makes it possible to generate alerts derived from the evolution of a patient’s chronic wound, by means of the interpretation and combination of data coming from both an image of the wound, and the considerations of a healthcare professional with expertise in the subject matter. Some positive results are already shown regarding the determination of the ulcer’s status in the tests that have been carried out so far. Therefore, it is considered that the proposed methodology might lead to substantial improvements regarding both the treatment’s efficiency and cost savings.

## 1. Introduction

Chronic wounds, and more specifically pressure ulcers (PU), are nowadays a severe problem for society because of the high expenses involved [1,2]. Those costs are mostly related to the activities for monitoring and assessing the effectiveness of the medical treatment provided to the patient.

Consequently, a trend towards treating PU at the patients’ homes has been developing in these last few years, looking to lower the treatment expenses [2]. Additionally, it is commonly accepted that there is a wide range of variability in the training level of the health professionals involved in the prevention and treatment of PU, leading, ultimately, to a lack of experts in this clinical field [3]. All these circumstances point to the need for developing specific tools to help to manage, remotely and effectively, the process for monitoring the treatment and curation of PU through the generation of early and automatic alerts associated to the risk factors that are commonly present in the negative evolution of this kind of skin wounds.

The use of applications based on remote medicine or ‘telemedicine’ [4,5,6,7,8,9,10,11,12,13,14,15] becomes useful in this context. Nowadays, these applications are already widely used to help healthcare services to follow up, assess, and diagnose patient conditions remotely. The recent use of decision support methods in the telemedicine field has significantly increased their preventive efficiency in the monitoring of medical treatments [16]. Great technological advances, together with novel protocols for information control and processing, are facilitators for a continuous improvement in the field, thus contributing to accelerate the decision-making process through the incorporation of expert systems (ES). Expert systems make it possible to draw conclusions and to infer decisions based on the information related to the patients’ health status exchanged between them and the healthcare services [17].

In this paper, a new methodology is proposed and designed, aiming to the management, monitoring and analysis of the process for the treatment and curation of PU. Starting from the input information coming from different qualitative and/or quantitative sources, the methodology makes it possible to generate alerts related to the ulcers’ healing process, so that any cases where it is necessary to recommend and define new or additional treatments are identified promptly. To that purpose, a system combining MATLAB^©^ (R2020a, MathWorks^©^, Natick, MA, USA) image processing algorithms with a decision management module supported by concurrent fuzzy inference engines was proposed. Such a decision management system incorporates autonomous decision-making concepts, supported by the definition of expert systems based on fuzzy logic, which makes it possible to generate alerts about the risk level of the chronic wound getting worse. Thus, it makes it possible to decide on whether the care provided to the patient’s wound is suitable to achieve its complete healing. At the same time that the quality and efficiency of the treatment are improved, with the corresponding improvement in the patient’s health, a reduction in treatment mistakes, length, and associated costs will be achieved.

This work is organized and developed across five sections. In the current Section 1, some concepts are introduced that are necessary to establish the conceptual grounds on which the proposed methodology stands. In Section 2, an introductory description to the design and definition of the methodology is made, establishing the different stakeholders, stages, and events involved. Additionally, the methodology is established and proposed, explaining all the details that are required to fully understand it. In Section 3, the methodology is applied to a practical case and the results are collected. In Section 4, the results obtained after carrying out this work are discussed. Finally, in Section 5, the most notable conclusions are presented.

### 1.1. Related Concepts

#### 1.1.1. Health Assessment Process for Pressure Ulcers

The evaluation of a PU can be defined in a generic way as a systematic process made up of three different stages:

- Gathering information about the patient and her wound.
- Evaluation of the collected information: patient’s potential risk factors, such as the stage in which the chronic wound is classified, its size, whether it presents exudate or infection, among others [18].
- Development of a treatment plan.

It can be seen that the process for collecting the information is vital for the correct assessment of the PU, since it constitutes the basic element from which the opinions of the Wound Care Unit expert are generated, and therefore, from which the optimal treatment plan is selected.

A source of information that must be highlighted here is the patient’s health history, since it shows a description of the patient’s health status to date (for example, information about previous pathologies, ongoing treatments or disabilities).

##### Scales for Risk Assessment of PU

It is clear that, because of its impact on the patient’s health, the assessment of the risk of developing a pressure ulcer is a measure of great relevance. Several different risk indicators have been defined in the medical literature that combine diverse information on the patient, making it possible to develop an overall impression of the patient’s general health status, and consequently, helping to decide on the actuations that are necessary to prevent the potential formation of PU [19,20]. The factors involved in defining these risk indicators are aimed towards, not only preventing skin wounds, but also the integral care to the patient [19]. That is why taking into consideration this kind of indicators may also be helpful in the stages of monitoring and assessing PU, making it possible to establish different patient profiles, and, as a consequence, to make apparent the most severe cases. Three different evaluation scales are generally used for that purpose [19]: the Norton scale [21], the Waterlow scale [21], and the Braden scale [21], this last being the most widely used [22], and therefore the one that has been selected for this work. It must be mentioned that, while the Braden scale is acceptable to identify the risk of PU formation when the patient is being given care at home, its predictive power is limited [20].

#### 1.1.2. Use of Expert Systems in Decision-Support Tools 

Within the framework of information systems (IS), it is possible to define and understand these as tools oriented to support the decision-making process. These systems, conceptualized as decision support systems (DSS), evolved rapidly in the past decades to become a discipline in themselves within the IS field, because of their flexibility and adaptability [23,24,25,26]. This versatility is precisely what allows the DSS to be multidisciplinary tools, and to embed different information management methods and techniques in their usage. Different architectures emerged within the DSS field, the most relevant being those that take advantage of the advances in artificial intelligence techniques to constitute true expert systems, capable not only of leading human decision-making processes, but also of achieving in themselves the ability to solve problems in real environments. Expert systems incorporate abilities to solve problems and to merge the knowledge of several different experts, and also the capabilities needed to enhance the DSS-supported decision process [27].

In this sense, some of the leading exponents in the DSS field are the ES-based decision support tools, developed since the 1960s [28,29,30], which make it possible to transfer human knowledge and experience to computers [30,31,32]. Systems are obtained in this way that have the ability to provide expert solutions to complex problems. As a result of the versatility and power of these systems in the resolution of real-world problems, they are frequently used in the fields of decision theory and information systems in a role aiming to complement decision support tools [33,34,35,36,37,38].

In essence, for the definition of an expert system, it will be necessary to start from an expert-knowledge base, a human-machine interface, an inference system, and the problem’s dataset corresponding to a real domain [28,29,31,32,39,40,41]. The last few decades have seen the emergence of decision support methodologies based on ES, grounded on different techniques and approaches [30], which allow the representation of the previously mentioned elements. As a result of their relevance in the field, we must highlight the rule-based systems [42], the neural networks [43], and the fuzzy logic inference systems [44,45], among other methodologies.

With all of the above, it is possible to approach the design process within the field of IS according to the scope defined by Hevner [26]. In his work, design science research is defined [26,46], applying its conceptual structure to build artifacts supported by software that allow to solve human problems through the use of ES. This is what is proposed in this article: the design of a methodology based on decision management that is capable of solving a problem in a real environment, which, in this case, will be the monitoring and assessment of PU.

As also happens in the traditional engineering-related applications, designing within the information systems field involves a conceptual and methodological process that starts with the identification of a problem and the corresponding needs to be fulfilled, and then evolves successively until a product or a final artifact is obtained that meets the requirements and constraints of the initial problem [26,46]. In this way, designing within the information systems framework will consist of, as already mentioned, obtaining a software-supported device capable of solving a set of problems that can be addressed by means of the handling and management of the information related to them. Additionally, and after the generation of the artifact, Hevner et al. proposed a set of guidelines or principles leading to the evaluation of the design in itself of the artifact.

#### 1.1.3. Expert Systems in Health Applications

In this point, the application of expert systems to the healthcare field is analyzed. First, it is necessary to contemplate if the healthcare field can be considered a field to which it is suitable to apply information systems. The answer is self-evident and immediate, because a healthcare organization, just like any standard business organization, needs to incorporate information systems and technologies for its everyday work management effort. Therefore, any healthcare control system, operating face-to-face and/or based on telemedicine, constitutes in itself an information system, in which information collection and processing functionalities are established, with the final goal of making decisions regarding diagnosis, treatments, or other matters [16,47].

It can be seen that a clinical evaluation process follows the same schematic pattern than a standard decision-making process. Firstly, information is collected, to be later processed and evaluated according to certain criteria, and finally, some actions are defined based on the conclusions previously obtained. Therefore, it is correct to model the issues in the healthcare framework that are part of an information system, with expert systems that collect information from the environment, process this information and generate a set of decisions involving a plausible and consistent resolution to the problem posed according to the accepted medical thought process.

There are already multiple applications of expert systems in the healthcare field at this moment. Regarding skin care in particular, the work of Hyubgjin et al. aims to analyze the accuracy of a system based on telemedicine for assessing the status of chronic wounds [48]. In order to carry out the assessment, digital photos, and other data from the wound and from the patient are collected on a laptop. That information is later uploaded to a webpage, allowing the experts to access it for assessing the wounds’ status. The potential of the system has been shown, making possible to improve the access to this service to people who are currently not being treated by specialized staff. Furthermore, Dimitrios and Fotini considered that the use of PU digital images processed with the help of image processing techniques may be helpful to classify the different regions that are present in these wounds [49]. In their paper, they proposed a tool that might help to determine the stage the PU is in at any time. That tool has two operation modes: the training mode and the operation mode. In the training mode, the tool is trained using several datasets coming from segmented images (their color and texture features including the labels established by an expert), in order to obtain the classifier parameters. In the operation mode, the tool uses these previously established classifier parameters to perform the classification of new cases. In the work of Julie et al., a web-based system using telemedicine tools for the evaluation of wounds was presented and described [50]. Such a system makes it possible to upload and consult digital images of the patient’s PU and other information that is required to determine the patient’s condition. Several other studies have been found in this field of work, aiming to evaluate the effectiveness of different telemedicine-based methods to assess the status of chronic wounds [2,51,52,53,54,55], with promising results overall. In the paper by Margaret Terry et al. [2], a study within the telemedicine framework was carried out to measure the effectiveness of using digital cameras for the treatment of patients’ wounds at home care. The authors were not able to prove that telemedicine was effective with their study design, but they believed that the use of these kind of telemedicine-based approaches for the treatment of wounds is still evolving. In the paper by Ling Jia Goh and Xiaoli Zhu [51], a review was presented, aiming to evaluate whether the use of telemedicine techniques could have a relevant effect on the improvement of the wounds’ treatment process, concluding that it may help to improve the wound healing rate. In the paper by Lauro S. Halstead et al. [52], a comparison was made between the remote assessment of PU in spinal-cord-injured patients using a simulated remote setting with face-to-face assessments. The information taken into account for such a remote assessment came from a digital image, plus the medical history and a wound-status form from the patient. The authors concluded that there is a significantly enough percentage of agreement between the remote and the in-person assessments outcomes. In the paper by Barbara Binder et al. [53], the feasibility and acceptance of telemedicine techniques applied to the wound care of patients with leg ulcers were analyzed. The authors concluded that there was an overall acceptance of these techniques by all the stakeholders involved: patients and healthcare staff. A reduction in costs and an improvement in the patients’ quality of life was also observed. The paper by Ralph Peter Braun et al. [54] analyzed the viability of performing remote medical wound care using mobile phones with integrated cameras. They concluded that such telemedicine approach may replace the in-person consultation in typical situations. The paper by R. Hofmann-Wellenhof et al. studied the viability and acceptance of the telemedicine approach in the Dermatology Unit for managing patients suffering with chronic leg ulcers [55]. After the initial face-to-face visit by a wound expert to establish the patient’s ulcer condition, the next monitoring visits were carried out by nurses. In those weekly visits, the nurses took pictures of the wounds and gathered other relevant information about the patient, all of that to be sent through a specific website to an expert belonging to the Wound Care Unit, who then assessed the wound status and stablished therapeutic recommendations. The authors concluded that there was a high degree of acceptation by all the stakeholders involved (patients and healthcare staff).

## 2. Materials and Methods

### 2.1. Definition of the Methodology

#### 2.1.1. Conceptual Design of the Proposed Methodology

In this work, the definition of a methodology is proposed, supported by expert systems applied to the diagnosis and treatment processes of PU, and focused specifically on their monitoring and assessment stages. The use of expert systems provides the methodology with the ability to solve problems and issues associated with the aforementioned processes, in much the same way as a team of healthcare professionals would do it.

It has been already mentioned that the design of the proposed methodology is formulated as the design of an artifact supported by software that fulfils the requirements and restrictions derived from the problem to be solved. Therefore, the design process must necessarily begin with a first stage of information retrieval, so that the definition of the needs associated with the use of the device and the functionalities that will allow it to solve the problem can be determined from such information. In this case, the data fed into the information system is obtained either by direct upload from the devices or derived from the healthcare assessment operation. After that, the specific technical requirements corresponding to the aforementioned needs will be elaborated, taking into account the set of restrictions of the problem. Table 1 summarizes those lists of needs, requirements and restrictions, based on the statement of the general purpose of the artifact.

**Figure 1:**
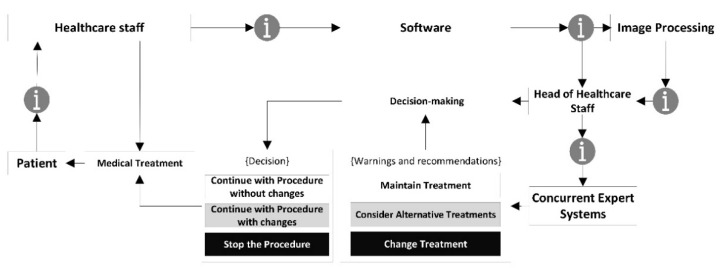
Conceptual schematic of the artifact showing the information flows.

A conceptual schematic of the artifact is to be developed from the list of needs as shown in Figure 1, including all the formal and procedural aspects necessary to achieve its goals. Figure 1 shows the information flow from its collection to its treatment in the expert systems, going through the different stages in the healthcare assessment operations. Following the contents of Table 1, the initial data about the patient’s condition is transferred through the software, complemented with an image showing the current state of the wound. All this information is analyzed by two concurrent expert systems that consider both the information from the system itself and the health assessment made on that information. This makes possible to obtain, firstly an initial decision as an output of the expert systems, and secondly a re-interpretation of that decision made by the healthcare team. 

Therefore, it will be necessary to establish the way in which the methodology will manage the information, and also to state which protocols to follow to collect, process and manage information. Additionally, the rules and behaviors that will handle decisions are to be defined, proposing an inference engine that incorporates them to support the decision process. Finally, an interface will be created to make visible all the information, the inputs and outputs necessary for the correct application of the developed methodology.

#### 2.1.2. Previous Considerations

As introduced before, a new methodology is designed and defined in this paper based on the use of expert systems, to be used in the monitoring and assessment processes of the medical treatment of PU. Furthermore, this methodology could generate alerts related to the PU status for each patient in particular. It will also provide support for the resolution of issues and questions related to the decision-making process associated to the evolution of the current treatment, through the analysis of the wound and the choice offer, if appropriate, for a new treatment. As it has already been justified in Section 1, the conceptual framework of the proposed methodology is the information system working towards the design of an artifact according to design science research. Prior to all this, and in order to validate the device presented, the true core model of the methodology, some guidelines and principles established by Hevner et al. [26,46] are to be followed. The seven guidelines proposed are a reference for evaluating the origin, adaptation and usefulness of the device within the design science, and therefore the IS, fields. The compliance of the presented methodology, represented by the software-supported artifact, to these guidelines will help to verify and justify its relevance.

Thus, prior to the detailed definition of the methodology, it is necessary to analyze the adaptation of such methodology to the guidelines established by Hevner et al. [26,46]:

- Guideline 1—Design an artifact: the proposed methodology, developed through a software-supported artifact that implements an expert systems, is presented in detail throughout Section 2. Its purpose is to help the healthcare team in the difficult stages of monitoring and assessing the status of PU. To do this, first the artifact supported by specifically developed software performs the calculation of two index values named ‘Technical Risk’ and ‘Expert Risk’. These are obtained as a result of processing the input information by means of two fuzzy inference engines working concurrently. Subsequently, these calculated risk indices are combined in order to produce an overall risk indicator called ‘Global Risk’, which allows to establish the risk level related to the non-improvement of the ulcer status with the current treatment. Finally, different recommendations can be established based on the interpretation of the Global Risk, as a result of which it might be necessary to immediately change the treatment that the patient is currently receiving, and to choose a new one. To facilitate all the necessary calculations, the implementation of the artifact has been carried out in the shape of an application developed using the MATLAB^©^ software. This makes it possible to facilitate and systematize its usage as a way to show its viability.
- Guideline 2—Relevance of the problem: the design and definition of a methodology that allows to keep watch over the PU monitoring and assessment stages is a relevant fact in the healthcare field, not only because of the repercussion associated with the social factors (swiftness of treatment, satisfaction, costs reduction, etc.), but also due to the progress itself in the implementation and use of information technologies in the healthcare industry. On the other hand, in many cases it may happen either that the professional in charge of the treatment is not properly trained in the subject matter, or that it may not be the same person who performs all the treatment sessions, thus making it difficult to monitor and assess it. For all the above-mentioned reasons, the design of a methodology to be applied in the field of chronic wounds is considered sufficiently relevant to justify its development.
- Guideline 3—Design evaluation: Section 3 presents a case study that illustrates with examples how the methodology works. In addition to showing the operation of the software-supported artifact, in Section 4, the usefulness and effectiveness of the methodology for solving the target problem are analyzed.
- Guideline 4—Contributions to the field of research: the specific contributions to the field of decision support methodologies applied to the telemedicine scope have already been pointed out in Section 1, and are developed in more detail in Section 4 and Section 5 of this article.
- Guideline 5—Rigor in the research: the new methodology has been framed within the context of information systems and design science research, through the design of a software-supported device based on expert systems, with all of that being approached in Section 1. Furthermore, their mathematical developments are basically grounded on the concepts of fuzzy inference systems, commonly proven and acknowledged, given their effectiveness and capability of managing the uncertainty level associated with any decision-making process.
- Guideline 6—Design as a search: in Section 1 a description of the context in which the proposed methodology is framed has been carried out.
- Guideline 7—Communication of the research: in Section 4 of this work, the contributions of the method and some future lines of work are presented. Likewise, Section 2 and Section 3 describe the new methodology in detail.

#### 2.1.3. Description of the Methodology

**Figure 2:**
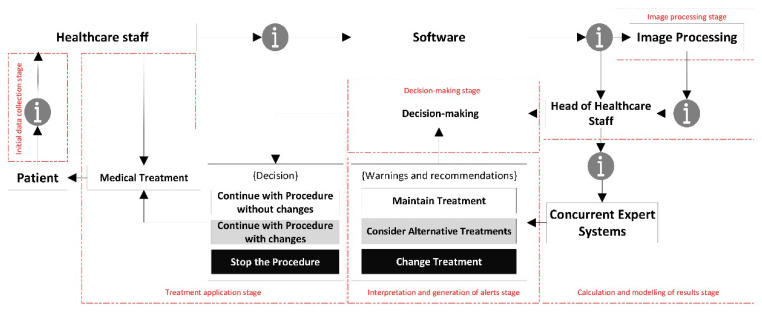
Stages of action in the proposed methodology.

Once the methodology has been assessed according to the design science guidelines, and its suitability and relevance in the field of application have been certified, it is necessary to proceed to describe it in detail. The proposed methodology is applied to the PU treatment and healing process, focusing on the monitoring and assessment stages. For this, the methodology states a set of stages that are deployed sequentially allowing to supervise from information collection to decision support, including the processing and inference of alerts. Starting with the design concept, the flowchart in Figure 2 represents different stages of action, collection and/or processing of information handled by the methodology on its own. A description for each of these steps is given in the next points.

##### Initial Data Collection Stage

The first stage of the proposed methodology deals exclusively with the process of home/hospital visitation of the healthcare team to the patient in order to monitor and treat the PU, while at the same time collecting the input data needed for the system to operate. Such data will come from different sources: images captured by the camera of a mobile device and data entered manually by the healthcare team, for example. The entire collection of these data could be supported by a mobile application or a web service, the definition of which is not relevant for the explanation of the methodology.

Thus, once the healthcare team arrives to the patient’s home or to the room where the patient is hospitalized, first a photograph of the PU is taken using the camera of a mobile device. Then, a form is filled in with the data needed to calculate a specific value for the patient in the Braden scale, used here to assess the risk level of PU formation. This test evaluates different aspects of the patient’s condition such as: sensory perception, activity, mobility, nutritional status, skin exposure to moisture, friction, and risk of injury [56]. In addition, the expert may request with certain periodicity additional data about other risk factors that she considers of interest.

##### Image Processing Stage

Once the images have been collected, it is necessary to establish an action protocol that allows to extract information from them. For this, a procedure for the analysis and processing of the digital images of the PU will be used in order to determine their surface area, providing a quantitative measurement associated to each individual image, patient, and date of the sample. This procedure is composed of two steps:

- Pre-processing: the saturation plane of the digital image is selected, inverted, and subsequently filtered as many times as necessary.
- Segmentation: the image is segmented in order to isolate the wound region in order to subsequently calculate its surface area measured in pixels. It is important to say that this segmentation uses a reference pattern that must be pointed at by the user. Additionally, it is possible to establish a sensitivity parameter associated to the segmentation threshold.

Image processing has been carried out by the implementation of basic functions of MATLAB^©^’s own toolbox, known as the ‘Image Processing Toolbox’ [57], combined with a specific segmentation script developed by Dirk-Jan Kroon [58], which applies the region-growing segmentation method. No modifications have been made or proposed in this case regarding the image segmentation procedure, as the results obtained from the application tests were considered optimal. Likewise, the relevance of any potential modifications with regard to decisions derived from the application of the methodology is debatable, as will be seen later.

It should be noted that a reference element with a known size must be present in the images to enable performing the geometric measurement estimations of the ulcer, such as its total surface area. Thus, it will be possible to calculate the changes in the wound surface area per unit of time using the data obtained from the current image and comparing it with the data from the first image of the ulcer taken before the treatment started.

##### Calculation and Modelling of Results Stage

The next stage of the methodology deals with the processing of the data collected. These data are fed into two concurrent expert systems that use Mamdani fuzzy inference systems [59,60,61]. These will calculate the Technical Risk and Expert Risk values respectively, both of them related to the estimation of a Global Risk value associated to the risk of the ulcer status not improving. All this will be explained in depth in Section 2.2.

##### Interpretation and Generation of Alerts Stage

The proposed methodology considers the efficient supervision of the development of the monitoring and assessment stages in the PU treatment process, in addition to the generation of alerts if the reference values stated for that purpose are reached after the evaluation process. Once all the data have been collected, these are treated by the expert systems that will eventually generate an alert as a combination of their respective outputs. This alert can have, in principle, three different and mutually exclusive states:

- Continue with the current treatment: no additional actions are recommended, as the evolution of the PU healing does not require it.
- Consider alternative treatments: it is recommended to consider potential alternative treatments or therapies for the cure of PU to replace the current one.
- Change the treatment immediately: it is recommended to stop right away the treatment that the patient is currently being given for her PU and replace it with a different one.

Thus, the final outcome of the methodology is determined by the combination of risks established by two concurrent expert systems. It will consist of an alert derived from a decision inferred from the data collected and the health evaluations, that will be subject to modification by the healthcare team if required. In this case, three qualitative statements associated with a Global Risk value determined by the expert system were established. The healthcare team is allowed to modify the number of states by setting new thresholds associated with different ranges of the Global Risk value, if they see it fit.

In any case, when the resulting state is to ‘Continue with the current treatment’, the healthcare team will always have the chance to manually generate an alert, thus activating the state for changing the treatment immediately.

##### Decision Stage

Once the results have been obtained, the expert must proceed to make a decision regarding the PU treatment process. In this case, for example, the expert might be a specialist in the treatment and assessment of chronic wounds, or a healthcare team specialized in wound treatment. If the methodology has recommended either to consider alternative treatments or to change the treatment immediately, it will be necessary to assess the different alternatives, and to decide on which new treatment will specifically be applied by the healthcare team.

The methodology performs here the role of a decision support system, providing the team with all the needed information for that decision, processed and enriched with the suggestions and recommendations that could have been inferred by the expert systems.

##### Treatment Application Stage

This is the final action stage, in which the healthcare team applies the treatment that has been recommended by the system. It is important to remember that the three possible scenarios depending on the alerts described in the ‘Interpretation and generation of alerts stage’ point would be as follows:

- Continue with the procedure without changes: it is recommended to continue with the current treatment.
- Continue with the current treatment with changes: it is recommended to continue with the current treatment while considering alternative ones.
- Stop the procedure: it is recommended to interrupt the current treatment and start with a new one as recommended by the expert.

### 2.2. Implementation of the Methodology

The methodology presented in Section 2.1 considers different stages of action that span from the collection of information by the sanitary team, through the subsequent extraction of characteristics from that information (image processing, quantitative and qualitative data) and the combination of all data available and their processing through expert systems, reaching the final stages of interpretation, decision-making and application. Although the methodology encompasses all the stages described in Section 2.1, its implementation is basically limited to the ‘Image Processing’ and ‘Calculation and Modelling of Results’ stages. The software-supported artifact that will be described harvests and consolidates the information collected in the previous stages and acts as an interface to allow all data to be introduced. After that, it proceeds to the image processing in order to extract its features, and then to the actuation of the concurrent inference engines. Once the outcomes from these engines are available and the corresponding alerts have been generated, the last stages described will begin, in which the healthcare team and the expert together must assess the conclusions obtained from the expert system, and make the appropriate decisions to be put then into practice accordingly.

The developed artifact is explained in detail next, both from the point of view of its behavior and ways of action, and regarding its technical definition.

**Figure 3:**
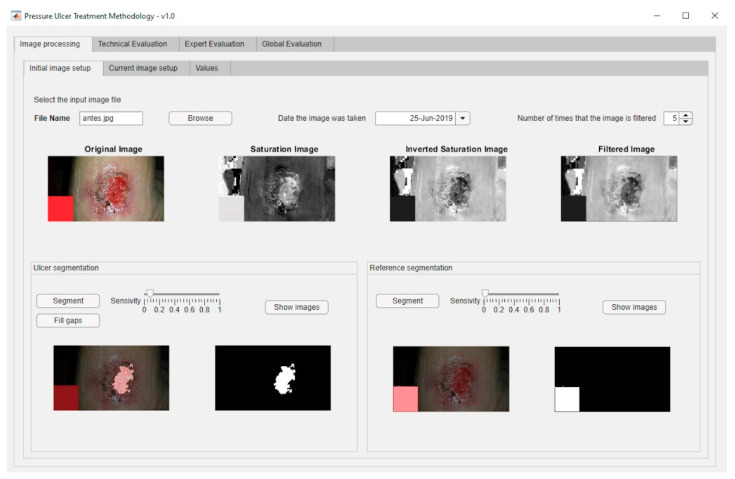
Dialog box for loading an image of the ulcer prior to the treatment.

**Figure 4:**
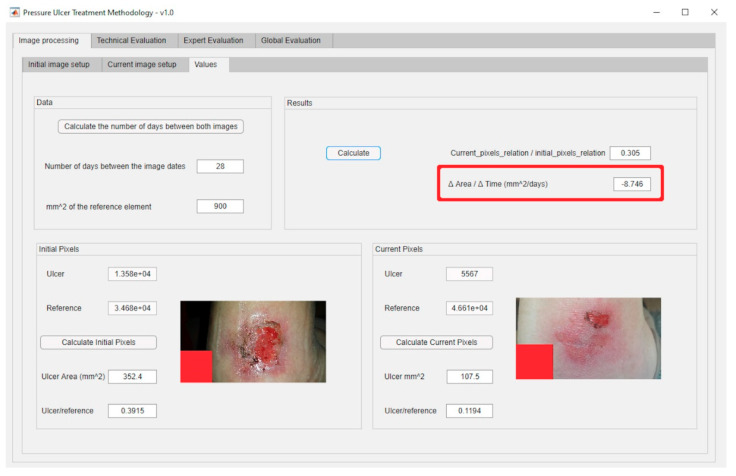
Dialog box showing the values obtained from the processing of the images.

From the ‘Initial Data Collection’ stage explained previously, it is necessary then to describe the ‘Image Processing’ stage. This stage involves imbuing into the artifact an image segmentation process based on MATLAB^©^’s Image Processing Toolbox [57], together with a specific segmentation script developed by Dirk-Jan Kroon [58]. This process consists of two steps: first a preliminary preparation of the image for the segmentation, and later the segmentation operation itself. Once an image is loaded, its saturation layer is then extracted and inverted based on the HSV (hue, saturation, and value) color model, and after that the image is filtered a number of times, five by default. Finally, the segmentation process is carried out (in this case, segmentation by growing of regions) from a starting point defined by the user. The execution and control of the process is carried out through an interface specifically created for this purpose that allows the user, in this case the healthcare team or expert, to upload the image, display it, and process it adequately. After that, the relevant image parameters are obtained, specifically the ratio between the change in the surface area of the ulcer and the time passed since the last picture. Figure 3 shows a dialog box for loading an image of the ulcer taken right before the treatment. This is an example image where the aforementioned reference element with known size is shown as a red square shape. Regarding the dialog box for loading the present-time images, it is very similar to the previous one. Furthermore, Figure 4 shows the dialog box displaying the values obtained from the processing of the images. The red box highlights the results of calculating the ratio of the change in the surface area of the wound to the time elapsed between the dates when the current image and the reference image were captured.

**Figure 5:**
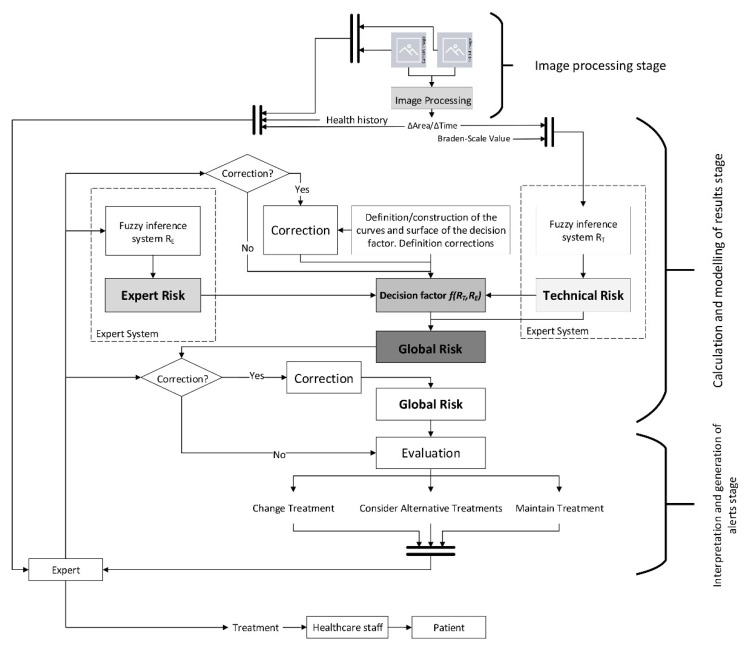
Flow chart of the system’s operation.

After this stage, the work progresses to the ‘Calculation and Modelling of Results’ stage. The artifact, supported by the designed software, makes possible to store and structure the data to be processed by a DSS defined in two steps. The first one includes an expert system focused on the management of the technical data derived from the image and from the specific Braden-scale value obtained. The second step involves another expert system that works concurrently with the first, and it is based on the criteria and the justified opinions of the healthcare expert after her assessment of the input data. The combination of both systems will finally produce a health alert level that corresponds to the Global Risk level of the ulcer status not improving, and that will require to be interpreted according to three possible states: continuing with the current treatment, considering alternative treatments, and changing the treatment. Afterwards, the expert will evaluate this information and, if necessary, will assess and select an alternative treatment. Finally, the said information will be transmitted to the healthcare team, which will be in charge of treating the patient according to the instructions sent by the expert. Figure 5 shows a flow chart that represents the overall behavior of the system.

#### 2.2.1. Technical Risk and Expert Risk Concepts

As shown in Figure 5, the Technical Risk value is obtained as the output of the first expert system. This system is in charge of evaluating the technical information, in this case the value obtained from the Braden test and the ratio of the change in the surface area of the wound to the time elapsed between the dates when the current image and the reference image were captured. This Technical Risk value quantifies the potential danger to the patient’s health obtained from an analytic measurement of the quality and efficiency of the treatment she is undergoing. This measurement is based only on quantitative data requiring little interpretation, thus lowering its uncertainty level defined as the variability in the accuracy of these data.

As seen in Figure 5, the Expert Risk value is determined as the output of the second expert system, which is responsible for the processing of information of a qualitative nature demanding a high interpretation effort. This information comes from the expert’s interpretations on the input information, the expert in this case being a specialist in the treatment and assessment of chronic wounds. This risk value provides a measurement of the subjectivity in the judgment and assessment performed by the expert, associated to her qualitative assessment of the data collected both about the PU status and about the patient’s own status. This risk value assumes a greater uncertainty level as it will be seen below.

The first system processes data coming from measurements taken with instruments that are considered as reliable and accurate. Therefore, the data obtained hardly need to be interpreted and should be considered as trustworthy. However, the second system performs a processing on the assessments expressed by an expert. Human language is imprecise and vague, which brings together an increase in the uncertainty level of the process, and eventually of the resulting decision. In this case, uncertainty is to be understood as the inaccuracy in the specification of a measurement, or as the difficulty in obtaining a quantitative value from information based on human judgment. In order to limit and control the uncertainty level in the application of the methodology, in this work Mamdani inference systems based on fuzzy logic are used because of their proven effectiveness in managing uncertainty. Additionally, Mamdani systems allow the fuzzy models to be represented in a way that is consistent with the logic, establishing consequents that are fuzzy sets in themselves. Mamdani systems make it possible to manage the imprecision in human language [59,60,61], and make possible the translation of qualitative into quantitative assessments, while keeping an effective control of such change.

#### 2.2.2. Global Risk and Decision Factor Concepts

The aim of this methodology is to present a system capable to perform an appropriate supervision of the monitoring and assessment stages in the treatment and care processes of PU. As a result of this, the final result obtained from the proposed concurrent expert systems will be the Global Risk value associated to the non-improvement of the pressure ulcer status. This is a quantitative risk value associated with both the current wound and the patient status, taking into account their previous statuses. That value provides the expert with an element of judgment from which it is possible to determine if the treatments given to the patient are appropriate to achieve the complete healing of the PU or if, on the contrary, it will be necessary to modify them.

The definition of the Global Risk is presented in equation 1. Its value is obtained from the combination of the ‘Technical Risk’ ($R_{T}$) and ‘Expert Risk’ ($R_{E}$) parameters previously obtained from two Mamdani fuzzy concurrent systems [59,60,62].

(1) $$R_{G}\left(R_{T},R_{E}\right)\;\to \;R_{G}=\;R_{T}\cdot \frac{f\left(R_{T},R_{E}\right)}{10}\;\forall \;R_{E,}R_{T}\;\in \left[10,\;100\right]$$

On the other hand, the $f\left(R_{T},R_{E}\right)\;$ term represents a function of the Expert Risk, which will be named as ‘Decision Factor’. Its calculation also depends on the Technical Risk. It is important to note that the Technical Risk has a lower bound corresponding to a minimum value of 10. For this condition the Global Risk value will equal the Decision Factor value, meaning that the final decision will depend exclusively on the expert evaluation.

##### Inference Systems: Calculation of Expert and Technical Risk

**Figure 6:**
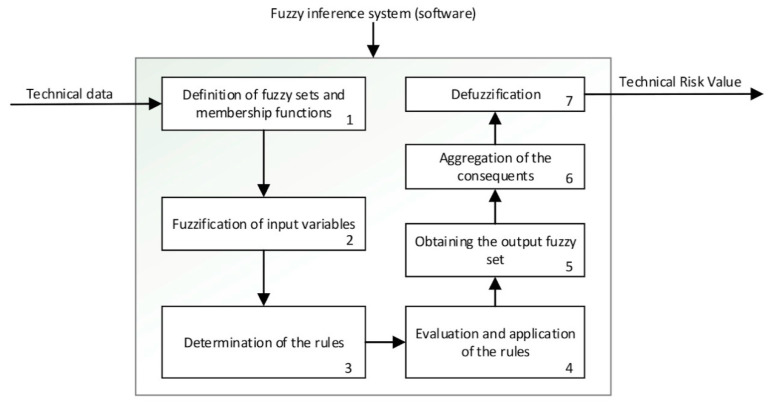
Diagram of the Technical Risk inference system operation.

**Figure 7:**
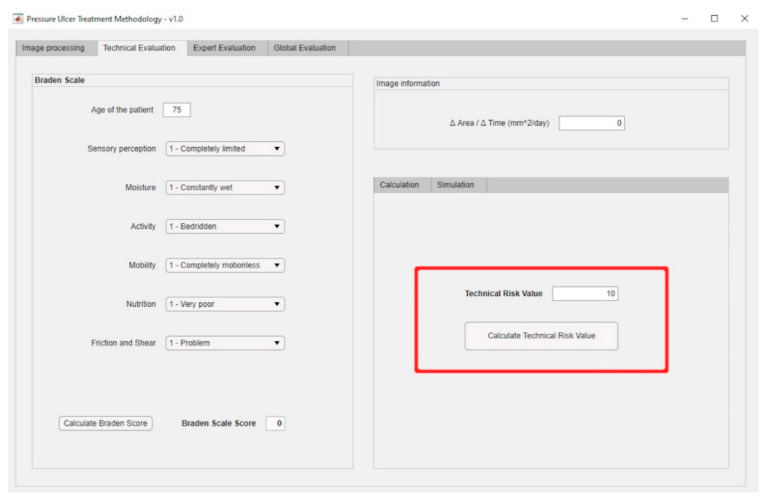
Technical Risk management panel.

**Figure 8:**
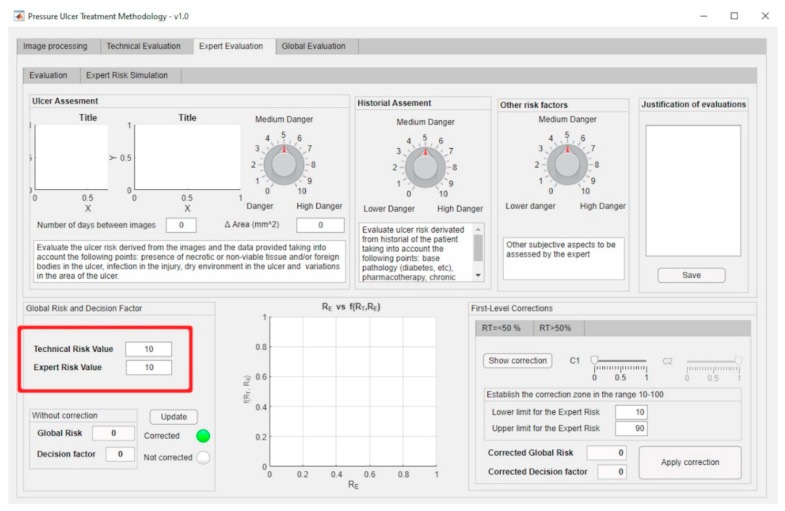
Expert Risk management panel.

The previously presented Technical and Expert Risk values are obtained as outcomes of the defuzzification process of their respective inference systems [62]. Figure 6 shows the schematic of the Technical Risk inference system, while Table 2 provides a description of its different stages. The Expert Risk inference system is very similar to the Technical Risk one. Likewise, Figure 7; Figure 8 show respectively the part of the interface that is in charge of supporting the inference engines in the case of the Technical Risk and in the case of the Expert Risk, with these values highlighted by the red boxes.

##### Calculation and Modelling of the Decision Factor

The value obtained from the first inference system, i.e., the Technical Risk, may be used directly because it is a non-interpretation-required parameter as it is derived from the input variables and obtained from measurements considered as accurate. On the contrary, the outcome of the second inference system, i.e., the Expert Risk value, is a parameter that needs to be interpreted, since it is derived from the inference process performed on the expert’s assessments.

Some degree of uncertainty in the determination of the Technical Risk can be accepted a priori, despite which, it will be considered in this work as an accurate value, because it is derived from reliable information. Even so, the inference system used to calculate that Technical Risk value incorporates an uncertainty control system inside, associated with potential flaws in the values fed to the system. That is why the Technical Risk will not be by itself decisive, but it will be placed in turn under the supervision of an expert who can assess the accuracy and reliability levels of the measurements. This must be done in this way, because a fully autonomous decision-making process is not to be reasonably accepted, especially in the healthcare field. Thus, the expert is not only in charge of supplying the data fed into the second inference system from which the Expert Risk value is obtained, but she must also carry out a qualitative assessment of the results derived from the calculation of the Technical Risk value. As a result of this, the influence of the Expert Risk on the Global Risk will depend on the Technical Risk value.

The effect of the expert’s decision in the methodology is represented firstly through the Expert Risk and later through the Decision Factor for the calculation of the Global Risk, and it must be consistent with the Technical Risk value, in order to avoid a potential conditioning or distortion in the final decision.

As mentioned before, the Decision Factor is the element that, combined with the Technical Risk, allows the calculation of the Global Risk. That factor is a mathematical function with the Expert Risk and the Technical Risk values as parameters, which enables the final decision to take into account the expert’s judgment, together with the assessment derived from the Technical Risk. By setting a range of values for the Technical and the Expert Risk from 10 to 100 and an inflection point at 50, the following relationships may be defined:

- For Technical Risk values up to 50, the Decision Factor value must be high, in order to obtain a high Global Risk value. It is established that the value of the Decision Factor must grow rapidly as the Expert Risk value increases. This means that, when the Technical Risk values do not indicate a critical state, that is, when they do not support a change in the treatment, then it will be in the hands of the expert to raise the severity level of the ulcer status through her considerations, thus, leading to a high Global Risk value.
- For Technical Risk values above 50, the Decision Factor value is not required to be as high as in the former case in order to produce a high Global Risk value, so it will be possible to obtain high Global Risk figures with low Expert Risk values. That is why, in these kind of cases where the Technical Risk is a dominant factor, the ‘change treatment immediately’ state prevails over the expert’s valuation, and it will be required then that she proves that the ratings reducing the previously established alert level are appropriate.

Having said that, it is possible to consider mathematical functions to model the behavior that was previously established between the different risk parameters and the Decision Factor, such as:

- Exponential curves: these allow to model the relationship between the Technical Risk, the Expert Risk, and the Decision Factor values for Technical Risk values up to 50, since:
  ◦ The Decision Factor value is virtually unchanged before the exponential zone is reached.
  ◦ These curves grow rapidly in the exponential zone.
  ◦ The Decision Factor will be a function $f\left(R_{T},R_{E}\right)\;$ that depends on the Technical Risk functions ($f_{1}\left(R_{T}\right)$ and $f_{2}\left(R_{T}\right)$), as well as on the Technical Risk $R_{T}$ and the Expert Risk, $R_{E}$ values.

Equation (2) shows the formulation proposed for the exponential curves. The functions $f_{1}\left(R_{T}\right)\;$ and $f_{2}\left(R_{T}\right)\;$ are set up initially at the beginning of the process. Equations (3) and (4) show the expressions for $f_{1}\left(R_{T}\right)\;$ and $f_{2}\left(R_{T}\right)\;$ obtained after applying the generalized reduced gradient method [68] and which will be used in the practical case presented in Section 3.

(2) $$f\left(R_{T},R_{E}\right)=f_{1}{\left(R_{T}\right)}^{\left(R_{E}-f_{2}\left(R_{T}\right)\right)}+10\;\forall \;R_{E,}R_{T}\;\in \left[10,\;100\right]$$

(3) $$\;f_{1}\left(R_{T}\right)=-0.0015\cdot R_{T}+1.1391$$

(4) $$f_{2}\left(R_{T}\right)=-0.0085\cdot R_{T}{}^{2}+0.5092\cdot R_{T}+57.247$$

- Logarithmic curves: this type of curves allow to model the relationship between the Expert Risk and the Decision Factor values for Technical Risk values above 50, since:
  ◦ They show a high growth rate in the first section, that is, for Expert Risk values from 20 to 30, after which the growth rate decreases.
  ◦ The decision factor $f\left(R_{T},R_{E}\right)\;$ will be a function that depends on the Technical Risk functions ($f_{3}\left(R_{T}\right)$ and $f_{4}\left(R_{T}\right)$) as well as on the Technical Risk $R_{T}$ and the Expert Risk $R_{E}$ values.

In Equation (5), the proposed expression for the case of logarithmic curves is presented. With regard to $f_{3}\left(R_{T}\right)$ and $f_{4}\left(R_{T}\right)$, these are set up like in the previous case, by applying the generalized reduced gradient method [68]. The expressions thus obtained are shown in Equations (6) and (7).

(5) $$f\left(R_{T},R_{E}\right)=f_{3}\left(R_{T}\right)\cdot \ln\left(R_{E}\right)+f_{4}\left(R_{T}\right)\;\;\;\forall \;R_{E,}R_{T}\;\in \left[10,\;100\right]$$

(6) $$\;f_{3}\left(R_{T}\right)=215.3\cdot R_{T}{}^{-1.005}$$

(7) $$f_{4}\left(R_{T}\right)=-0.0048\cdot R_{T}+0.7849$$

**Figure 9:**
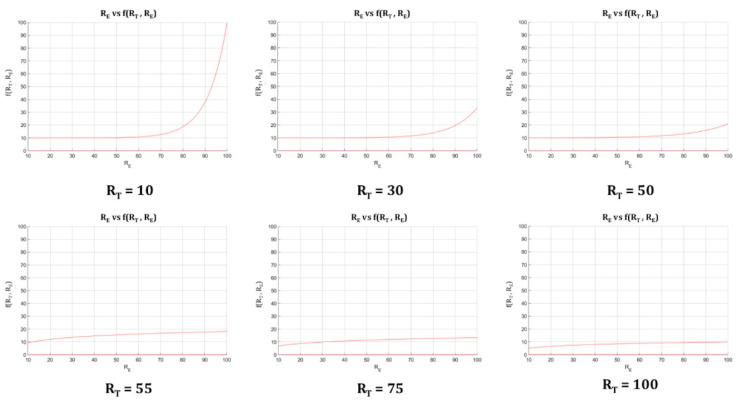
Fundamental curves: Expert Risk vs. Decision Factor for different Technical Risk values.

Figure 9 shows several curves in which the Expert Risk and the Decision Factor values are represented for different values of the Technical Risk.

**Figure 10:**
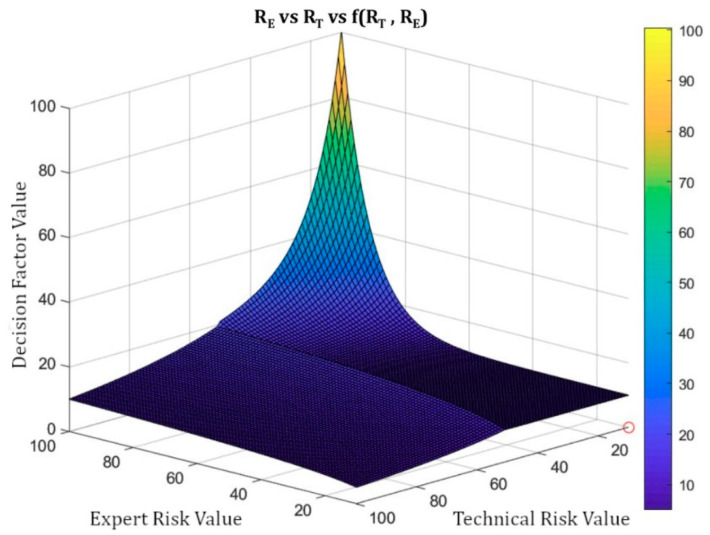
Technical Risk vs. Expert Risk vs Decision Factor.

It is possible to calculate the surface representing the Decision Factor versus the Technical and Expert Risks using Equation (2) through (7). Figure 10 shows the surface representing the Technical Risk and the Expert Risk versus the Decision Factor. It is possible then to notice those points where the trend or the growth of the Decision Factor are reversed, as well as the ones in which such trend or growth is more marked, aiming to suggest corrections and to prevent abnormal behaviors. The corrections described below make possible to correct the curves from a level above their definition, even when the artifact is in operation.

**Figure 11:**
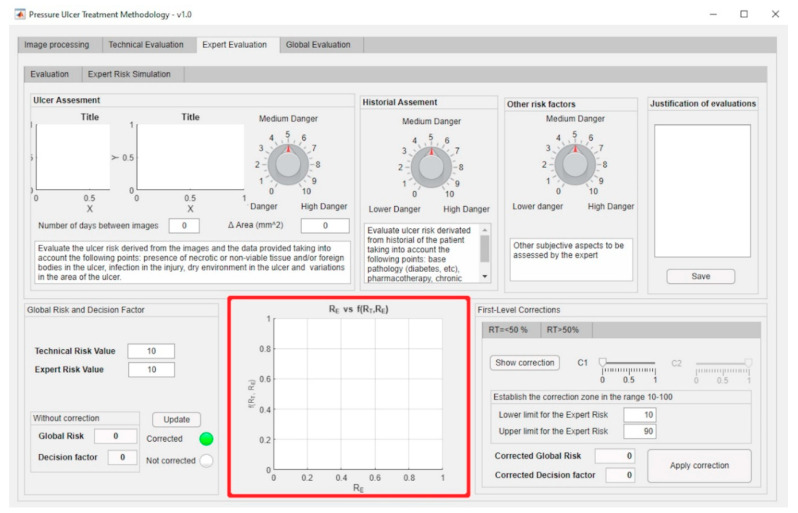
Panel for the definition of the curves’ model.

Figure 11 shows a screenshot of the interface where it is possible to recognize the parts of the application that implement the management and definition of the curve models as described before. The red box highlights the area where the fundamental curves (see Figure 9) are displayed.

##### First-Level Corrections

In order to adapt the process of calculating the Technical Risk and Expert Risk values to the observable reality by controlling the uncertainty of its autonomous determination, it has been resolved to provide the methodology with a tool to facilitate the correction of the results’ trend. This allows the expert to modify the default definition of the calculation model by means of a set of corrections, changing as a consequence the result finally obtained. The purpose of this process is, besides extending the usability of the methodology in itself, to increase the reliability of the decision made.

The first-level corrections aim to provide versatility to the methodology, since it might happen that the initial definition of the curves was not adapted to a specific case, and that is why the expert must be given the choice to increase or decrease the alert level obtained through the precise spot modification of some regions in the curves. These corrections can be applied both to the exponential and to the logarithmic zones.

##### First Level Correction-Exponential Zone

As previously indicated, the exponential zone is the region that encompasses Technical Risk values up to 50. It is characteristic of the exponential zone that the value of the Decision Factor function is practically constant until the fast-growing zone is reached. After that, it grows fast until reaching its maximum value. Given this property of the exponential curves, it might be the case that the expert wanted the function to start growing earlier, or else that it showed a higher growth rate than the exponential function. In order to achieve this, the expert is given the option to choose a range of Expert Risk values within which the behavior of the Decision Factor is to be modified.

**Figure 12:**
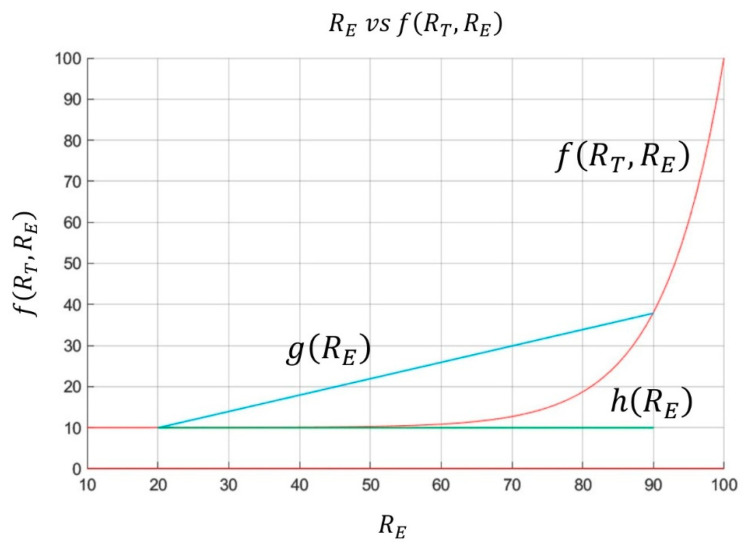
Expert Risk vs. Decision Factor.

Some auxiliary functions must be defined prior to the detailed definition of the correction. Figure 12 is taken as a reference to achieve this, showing the Decision Factor and two auxiliary segments. The upper segment is determined from the Expert Risk values established by the expert and the respective Decision Factor values corresponding to those points. The segment parallel to the abscissa axis is determined from the lower limit of the correction interval established by the expert and the value of the Decision Factor for that point. This segment’s length is determined by the difference between the upper and the lower limits previously established for the Expert Risk. Taking these definitions as a starting point, it is possible to define the differences d_1_ and d_2_ as shown in Equations (8) and (9).

(8) $$d_{1}=g\left(R_{E}\right)-f\left(R_{T},R_{E}\right)\;\forall \;R_{E}\;\in \left[10,\;100\right]$$

(9) $$d_{2}=f\left(R_{T},R_{E}\right)-h\left(R_{E}\right)\;\;\;\forall \;R_{E}\;\in \left[10,\;100\right]$$

The function that allows to calculate the corrected Decision Factor value can be formulated from the definitions established above, as shown in Equation (10). In this expression the expert must decide to which one of the differences to give priority, that is, which one will be more influencing through its weighting coefficients c_1_ and *c*_2_, coefficients that must obey Equation (11). It is relevant to mention that when choosing element d_1_ dominating over *d*_2,_ then a much more radical correction will be applied. Otherwise the correction will produce a much less marked change.

(10) $$f{\left(R_{T},R_{E}\right)}_{corrected}=f\left(R_{T},R_{E}\right)+d_{1}\cdot c_{1}+d_{2}\cdot c_{2}$$

(11) $$c_{1}+c_{2}=1$$

As the value of $f{\left(R_{T},R_{E}\right)}_{corrected}$ can be higher than $f\left(R_{T},R_{E}+1\right)$ at the right end of the correction interval, an upper limit for the corrected Decision Factor value should be applied, setting its maximum value equal to that established by the function $g\left(R_{E}\right)$ at the point. It was decided to take the value of the function at the point $\left(R_{T},R_{E}+1\right)$ as a reference to determine whether the value of the corrected function $f{\left(R_{T},R_{E}\right)}_{corrected}$ moves away from the value of $f\left(R_{T},R_{E}\right)$ at the right end of the interval. In the cases where the growth rate of the function in the correction interval is not significant, we could only make the comparison with the extreme value of $f\left(R_{T},R_{E}\right).$

##### First Level Correction-Logarithmic Zone

As explained above, the logarithmic zone contains those Technical Risk values greater than 50. In general, it is observed that for Expert Risk values in the interval [10,11,12,13,14,15,16,17,18,19,20,21,22,23,24,25,26,27,28,29,30,31,32,33,34,35,36,37,38,39,40] the Decision Factor values returned cause the Technical Risk value to be larger than the Global Risk value obtained. As a result of this, it has been considered to implement a correction to counteract the potential effect of under-valuations by the expert that could prevent a change of the PU treatment when she is not definitely certain about it.

For this, a decision was made so that when the percentage variations between the Technical Risk and the Global Risk values exceed a certain threshold value, for example 20%, then the expert will be asked about her level of certainty on the assessment made, and so the Decision Factor value will be corrected. Equation (12) is used to implement the mentioned correction within the logarithmic zone.

(12) $$f{\left(R_{T},R_{E}\right)}_{corrected}=\;f\left(R_{E}\right)\cdot \frac{100}{\%\;Security}$$

Finally, even if the corrected Decision Factor value might produce Global Risk values greater than 100, these values will never exceed this figure, which will be the upper limit of the correction.

**Figure 13:**
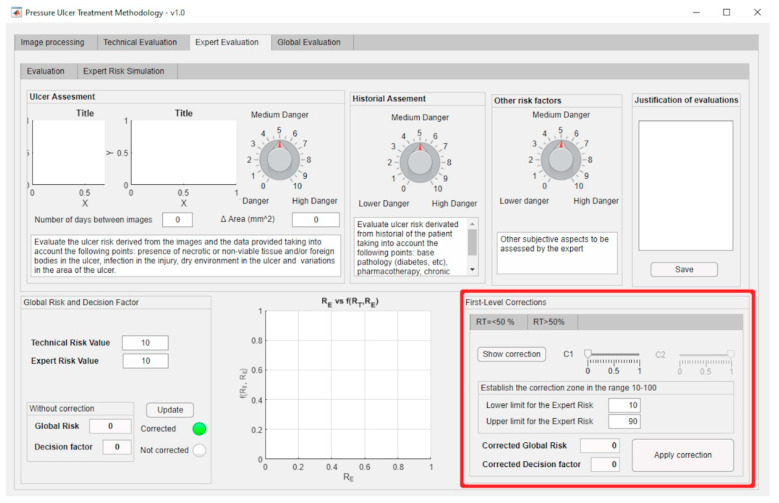
First-level corrections panel.

The first-level corrections, highlighted by the red box, for both the exponential and the logarithmic zones can be managed through the software-supported artifact interface shown in Figure 13.

#### 2.2.3. Determination of Global Risk and Alert Level

**Figure 14:**
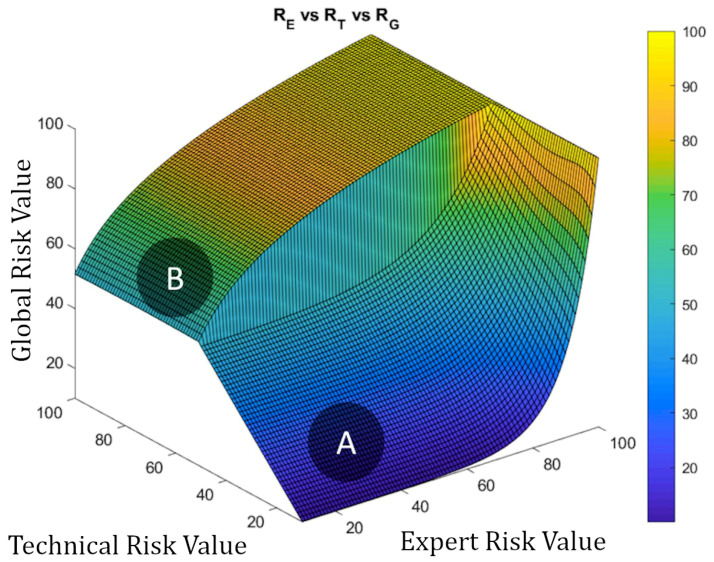
Global Risk surface.

Once the system has been defined, and the value of the Global Risk has been determined as a combination of the Technical Risk and the Expert Risk values using the previously presented Decision Factor, it is possible to construct the surface representing the Global Risk values versus the Technical Risk and Expert Risk values as shown in Figure 14. Two different zones can be appreciated in that figure: the first, labeled as ‘A’, corresponds to Global Risk values calculated by applying the exponential expression, while in the second, labeled as ‘B’, the logarithmic expression is used. As also happens in Figure 10, Figure 14 shows clearly the leaps and steps that usually happen in valuations, in this case of the Global Risk. This makes possible for the expert to analyze and evaluate the resulting image to decide whether she wants to use a new set of corrections to force adjustments in the behavior of the Risk curve.

**Figure 15:**
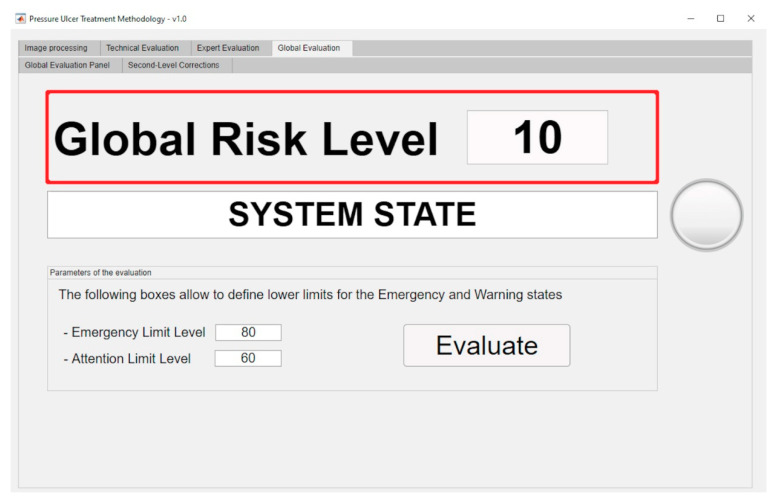
Global Risk calculation panel.

The aforementioned surface is used in the calculation of the final value for the Global Risk, whose dialog panel is shown in Figure 15. The red box highlights the final Global Risk Level.

##### Second-Level Corrections

As in the case of the first-level corrections, it might happen that the previously defined Global Risk surface (see Figure 14) does not model with accuracy the real decision-making scenario, thus, not satisfying the needs that potentially exist for each case. In order to overcome this possible limitation, it has been decided to implement a functionality to give the expert the choice on modifying the original surface by establishing a sigmoidal transition zone between the exponential and logarithmic areas. It is considered that this curve represents in a more appropriate way the transition between the patterns that have been observed. Any other transition model that could be considered would be easy to implement on the system.

A sigmoidal correction allows to establish a smooth transition zone, removing the sharp boundary between the exponential and logarithmic zones in the original graph. Even if this correction aims to get close to a usual distribution curve, the expert will always be on charge of assessing its adequacy and level, and also to either implement it if applicable or to void it.

In order to apply this correction, the expert must first define first the range of Technical Risk values $\left[R_{T1},R_{T2}\right]$ between which she wishes to perform such correction. It is required that the change value for the interval, i.e., 50, is contained within that range. In addition, the maximum value for the Technical Risk that could be selected cannot be higher than 75, while its minimum value cannot be smaller than 25. These limitations prevent the system from drifting away from the behavior previously established through the exponential and logarithmic functions. Equation (13) shows the expression for calculating the corrected Global Risk value for a specific value of the Technical Risk $R_{Tj}\in [R_{T1},R_{T2}]$ and for a certain value of the Expert Risk $R_{Ei}$. By modifying the parameter b in that equation, it is possible to open or close the sigmoid on the Technical Risk axis.

(13) $$R_{G\;corrected}\left(R_{Tj},R_{Ei}\right)=R_{G}\left(R_{T1},R_{Ei}\right)\;+\frac{\left[R_{G}\left(R_{T2},R_{Ei}\right)-R_{G}\left(R_{T1},R_{Ei}\right)\right]}{1+e^{-b\cdot \left[R_{Tj}-\frac{R_{T2}+R_{T1}}{2}\right]}}$$

**Figure 16:**
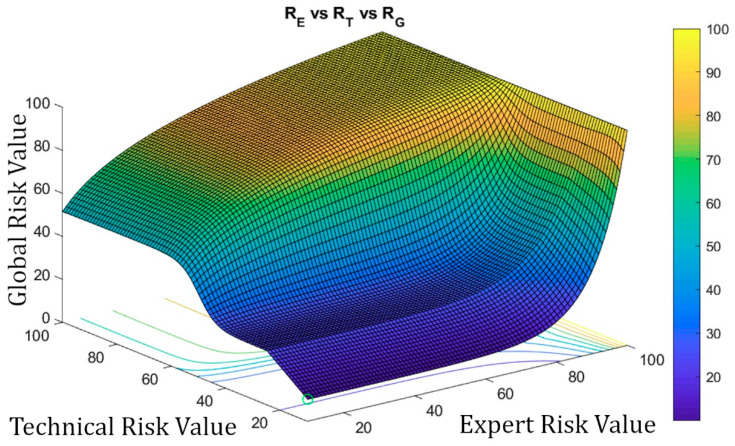
Technical Risk vs. Expert Risk vs. Corrected Global Risk.

In order to show the graphical outcomes of applying a sigmoidal correction, Figure 16 shows the results obtained after modifying (by applying Equation (13)) the initial plot shown in Figure 14 for Technical Risk values included in the interval 25–75.

It is important to stress that the application of a second-level correction is fully compatible with the application of a first-level correction.

**Figure 17:**
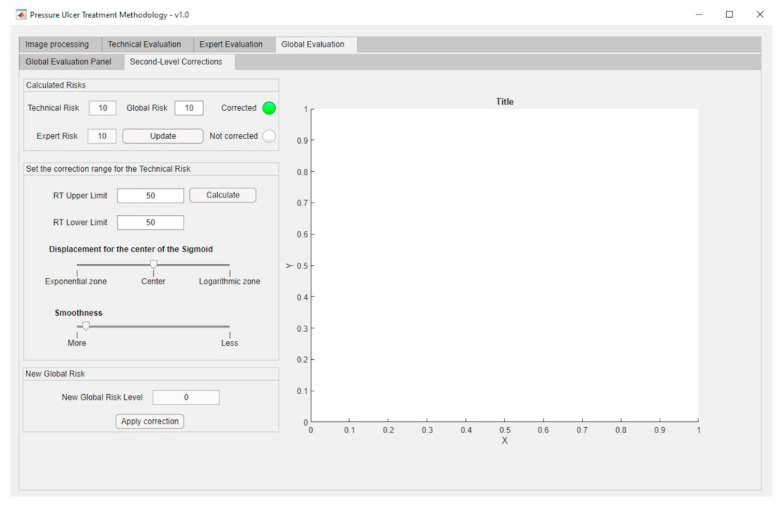
Second-level corrections panel.

Second-level corrections are accessible through the interface as shown in Figure 17.

#### 2.2.4. Treatment Evaluation

Once the Global Risk value has been calculated, the methodology elaborates the conclusions derived from the supervision of the monitoring and assessment stages of the PU’s treatment process. This Global Risk value makes possible to evaluate the effectiveness of the treatment through the interpretation and consideration of its results. Some intervals of the Global Risk have been already established (it is possible to change them at any time) in order to define the following states of the system:

- Continue with the current treatment: the Global Risk value lies within the range [0–60).
- Evaluate alternative treatments: the Global Risk value lies within the range [60–80).
- Change treatment immediately: the Global Risk value lies within the range [80–100]

From that evaluation above, the expert must communicate new instructions to the nursing team, for example, it might be necessary to select a new treatment to keep a positive evolution of the healing process in the pressure ulcer.

The expert (or the healthcare team) can re-evaluate the ranges associated with the alerts at any time, either at the beginning of the process or at any of the different iterations. This makes it possible, not only to extend or reduce those ranges, but also to increase or decrease the number of alert intervals.

In order to support the methodology, the software developed also allows to generate the aforementioned alerts, and to provide the suggestions showing them as a graphical and textual combination as seen in Figure 15. With this the software achieves the dimension of an effective decision support tool as it has been already pointed out.

#### 2.2.5. Implementation of the Artifact at Software Level

Once the methodology was designed and described, it has been implemented in software using the MATLAB^©^ R2020a platform’s Image Processing Toolbox [57] and the aforementioned script for image segmentation [58], implementing the inference systems by using the Fuzzy Logic Toolbox [69]. An integral interface was also developed using the App Designer module. MATLAB^©^ R2020a [70] provides a high level of versatility for the development of calculation tools and has demonstrated to be a robust platform for the development of inference systems.

## 3. Practical Example and Results

It this section, the application of the methodology on a practical case is presented. The initial case data was collected thanks to the support and collaboration of a healthcare team specialized in ​​wound treatment. All the privacy protocols have been followed along the whole process, and the case images may be distributed without harm to any person, collective or institution, since its use has been granted for training or academic research purposes.

### 3.1. Stage 1: Data Collection

As already pointed out, the initial data were collected by a healthcare team specialized in wound treatment in a Spanish hospital, and its details are presented in the next paragraph. 

**Figure 18:**
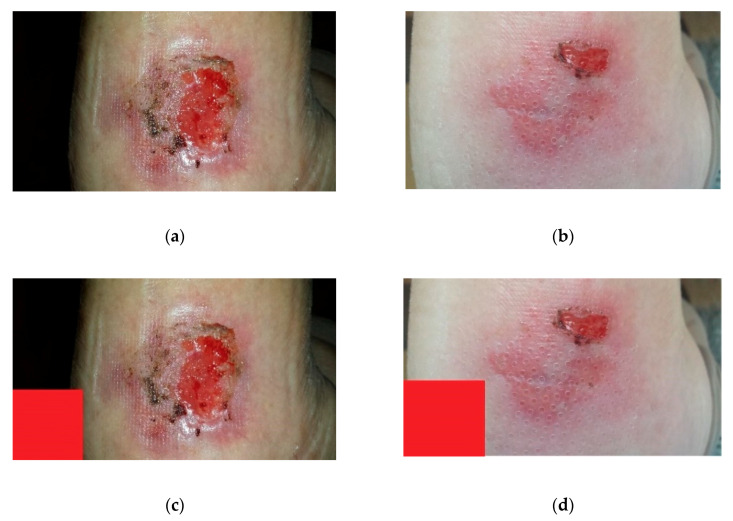
Photographs of the wounds: (**a**) initial picture; (**b**) current picture; (**c**) initial picture, modified; (**d**) current picture, modified.

A 94-year-old patient suffers a chronic wound as shown in image (a) of Figure 18 (image date: 25 June 2019). Image (b) of Figure 18 shows the status of the same wound 30 days later (image date: 23 July 2019).

To illustrate with an example the operation of the methodology, a virtual reference element has been manually inserted into the picture (in the actual application a physical element placed near the wound would be used as reference). Prior to that, the resolution of both images has been compared, and, taking into account the measurement of the apparent width of the leg, the aforementioned reference element has been sized and placed according to that proportion. See pictures (c) and (d) of Figure 18.

### 3.2. Stage 2: Image Processing

**Figure 19:**
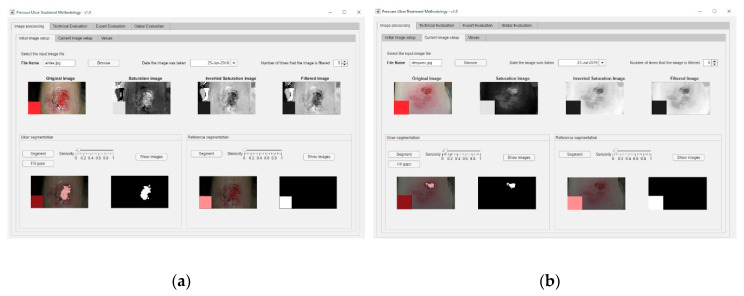
Image processing: (**a**) initial picture; (**b**) current picture.

**Figure 20:**
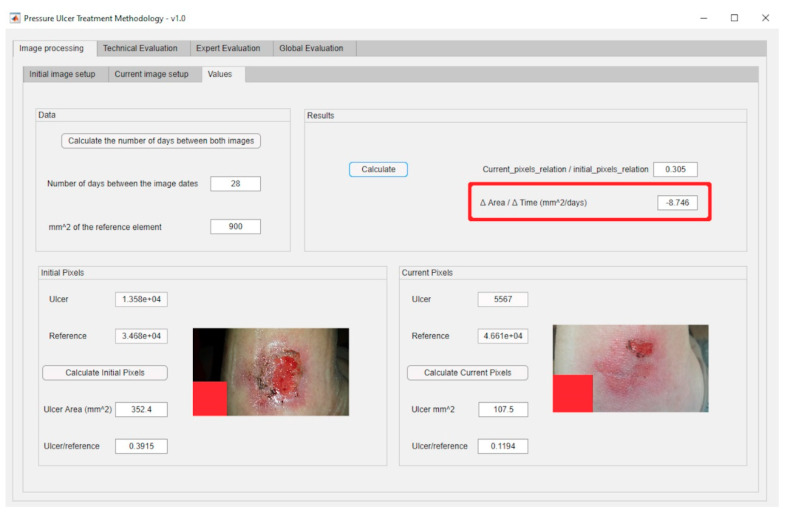
Output values of the image processing stage.

In order to carry out the image processing of the wound pictures, the process starts by processing the corresponding images of the wound in the initial and the current statuses. Figure 19a shows the dialog panel used for the processing of the wound’s initial image, while Figure 19b shows the panel used for the processing of the wound’s current image. From the values of the wound surface areas in both pictures it is possible to calculate subsequently the rate of change in the surface area versus the time elapsed between both image dates, factor represented as ΔArea/ΔTime, as seen in Figure 20.

### 3.3. Stage 3: Calculation and Modelling of Results

**Figure 21:**
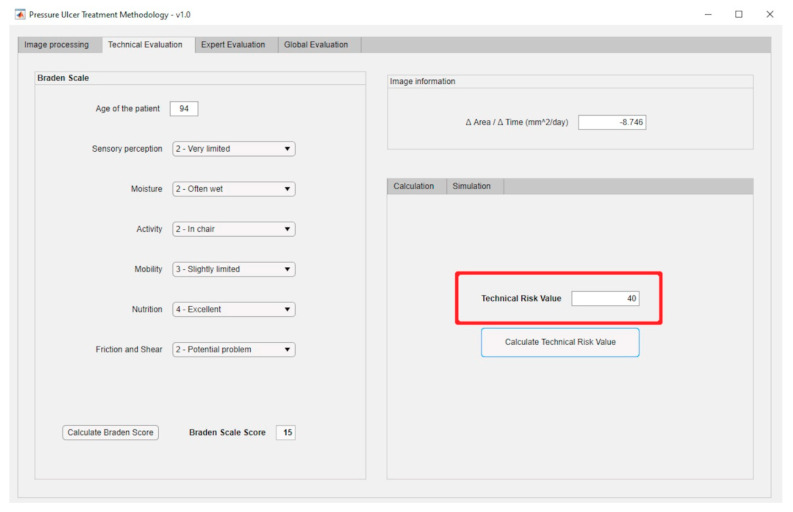
Technical evaluation panel.

In this stage the necessary data for the correct operation of the inference system are provided. The nursing team enters the assessment scores for the different Braden scale items (see Figure 21 for the Braden scale), and thus for this case a Technical Risk value of 40 is obtained.

Next, the expert evaluates the images, health history, and other risk factors, according to her judgment and experience. For this particular case:

- Evaluation of the images: she considered a slightly low risk level, since there is no necrotic tissue, foreign bodies or infection present, the ulcer environment is wet, and the wound surface area has decreased since the last picture (3/10).
- Assessing the patient’s health history: this is an elderly female patient with reduced mobility. She suffers diabetes that is currently undergoing treatment. It is therefore considered a potentially high risk (8/10).
- Other risk factors: a telephone interview with the caregiver finds that the patient has improved in her diet. It was also observed that, because of the wound status improvement, she is not in so much pain anymore and her mobility has improved (4/10).

**Figure 22:**
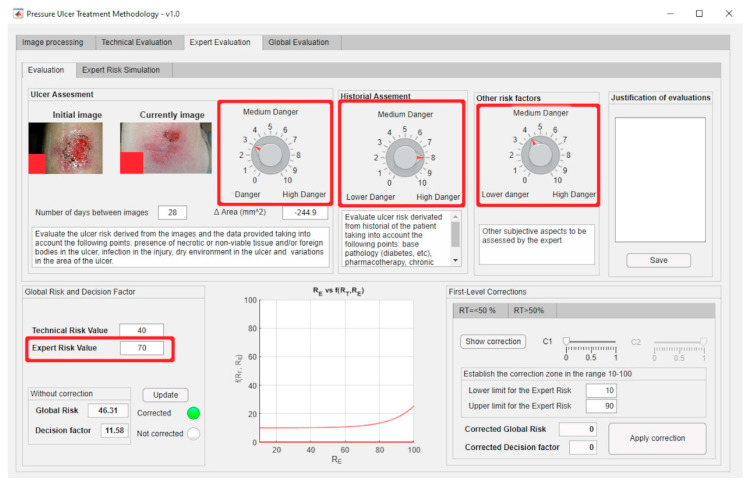
Expert evaluation panel.

The expert introduces said evaluations in the application and thus the corresponding Expert Risk value is obtained, in this case of 70 as seen in Figure 22 (please see in the upper part of the figure the assessment knobs highlighted by the red boxes).

A Decision Factor value of 11.58 is obtained from the pair [Technical Risk, Expert Risk], thus producing a Global Risk value of 46.31.

### 3.4. Stage 4: Interpretation of Results and Generation of Alerts

**Figure 23:**
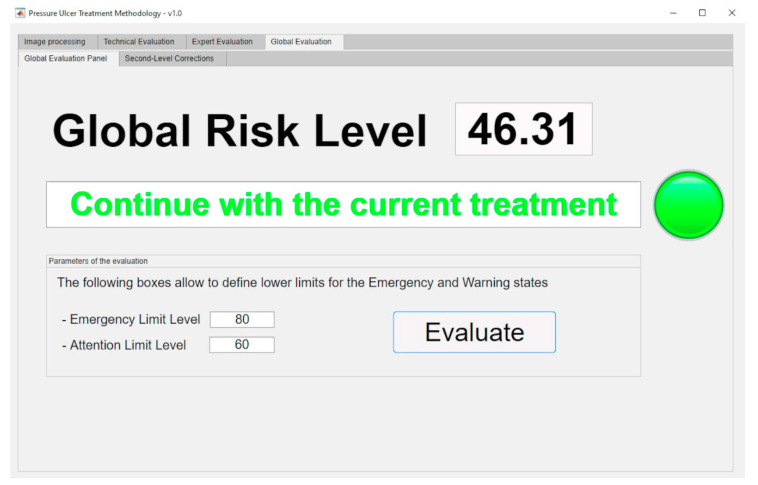
Evaluation panel.

The Global Risk value obtained without any corrections was initially 46.31, thus implying the recommendation to continue with the current treatment, as seen in the Figure 23.

### 3.5. Stage 5: Decision-Making

Once those first results were available, the expert healthcare team decided for this case to review the Risk values, as these seemed to be too conservative according to the patient’s state and evolution. They resolved to proceed with a series of corrections, as described next.

**Figure 24:**
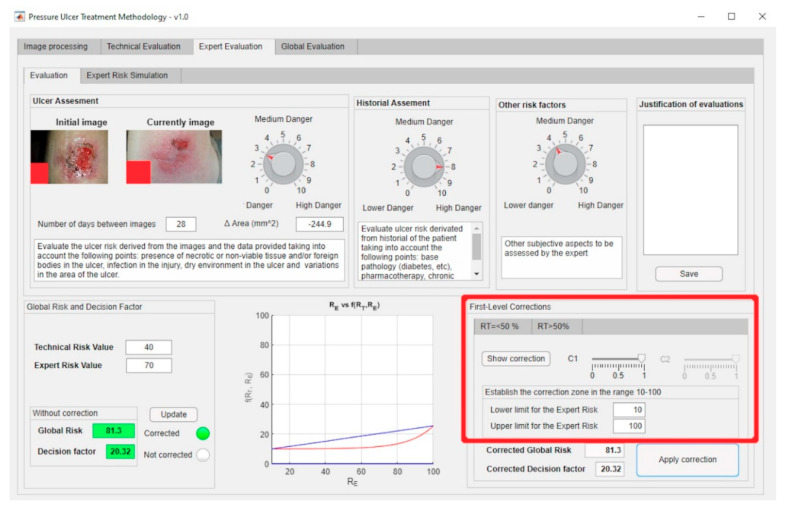
First-level correction.

The team decided to make a correction on the value of the Decision Factor. In this case, a range of Expert Risk values was established within which that value was to be modified, and the correction parameters were set. Thus, a corrected Decision Risk value of 20.32 was obtained. To perform such correction, the expert health team considered as not effective to continue with the current treatment because it was very demanding for the patient. It was deemed appropriate to cancel it and start another one compatible with outpatient treatment for completing the healing of the wound. Figure 24 shows the application of that correction to the Decision Factor curve.

**Figure 25:**
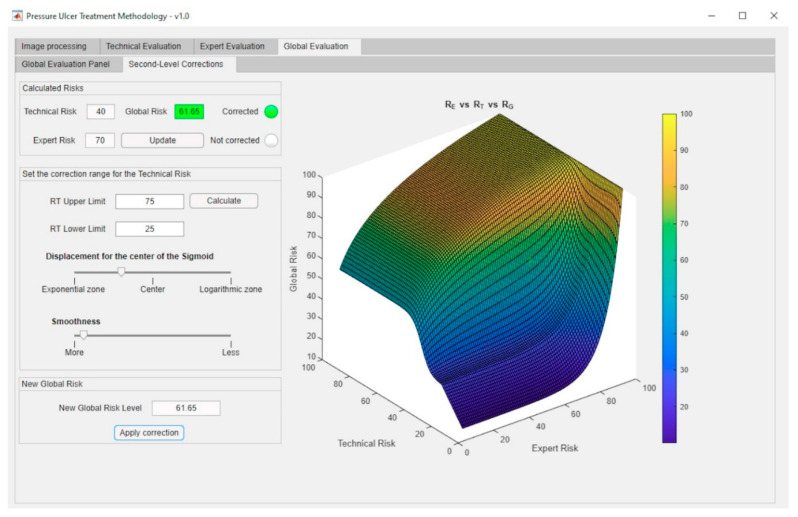
Second-level correction.

Similarly, a second level sigmoidal correction was applied to the Global Risk surface, arguing that the positive evolution of the wound was faster than the initial expectations. Figure 25 shows the dialog panel for defining these sigmoidal corrections.

After making these corrections, the health expert team assessed: first, that the initial treatment had been successful; and second, that, due to factors not related to the patient’s health, it was advisable to change to a similar treatment with a lower cost that allowed for an outpatient follow-up process.

### 3.6. Stage 6: Treatment Application

The decision-making process supported by the methodology led to the following outcomes:

- Although the patient had experienced an improvement, it was observed that the current treatment effects were clearly slowing down, therefore not justifying its cost, and its effectiveness was being jeopardized.
- A decision was made to switch the treatment to another one involving a lower cost but similar healing efficacy.

Although a long-term study has not been launched yet, the results obtained for this case study made it possible to gain confidence in the use of the developed software. Through the recommendations and the possibility to correct and to adjust it to different situations, this software can be adapted to changing environments, such as healthcare. After applying the aforementioned decisions, the patient’s condition showed a very positive evolution, resulting in the complete healing of the wound some weeks after this study was completed.

## 4. Discussion

The use of expert systems as computational tools capable of solving problems has evolved, from an initial conception linked to design support systems under the information systems framework, to its conformation and validation into methodologies that go further than problem solving: to do it in a way that mimics human thought. It is the implicit combination of information optimization and processing that provides expert systems with a great ability for modelling complex multi-criteria decision environments. Similarly, the development of software artifacts according to the recommendations of design science that integrate expert systems to formulate solutions to actual problems has seen its footprint and versatility increased. In this article, a methodology is designed precisely as an information systems artifact which solves an open problem in a complex environment using expert systems. These environments are not alien to the healthcare field, which is rather a representative of them. The disease diagnosis and monitoring processes are subject to endless decisions having a direct impact on the patient’s health, while also leading to inescapable economic consequences. This article presents a novel actuation methodology to be applied to the monitoring and assessment stages within the treatment process of a type of wounds known as ‘pressure ulcers’. By defining a sequence of stages and establishing actuation protocols along them, it is achieved to control the process of information collection and processing that, together with the use of concurrent inference engines, makes possible to produce recommendations related to the success and failure in the diagnosis and treatment of PU. This means that the methodology is provided, not only with the ability to manage the information collected along the process, but also with the capability to reduce its own uncertainty through a comprehensive supervision of the measurements collected and, above all, the supervision of all those operations that are subject to expert judgment.

The conceptualization of expert systems comprehends increasing the diversification of information sources, usually experts in an area of knowledge, in addition to providing a platform for the formalization of those sources [27]. In our case, when evaluating an ulcer, the healthcare team should look for the most reliable sources of information, that is, to reach for the assessment of the most prominent experts in the field. The proposed methodology allows the information to be captured through its interface, so that it can be evaluated by the expert no matter when or where she is. Likewise, it provides the appropriate formalization by translating all the qualitative information into quantitative and, either directly of in an inferred way, into interpreted values. It is clear that the use of expert systems substantially improves the treatment of wounds, and marks a milestone regarding the current treatment-support processes.

With regard to the outcomes of applying the methodology seen in Section 3, it must be stressed that, even if they show just isolated results, their validity can be assured by relying on the aforementioned condition: the adaptability of the methodology to the experts’ criteria. The example shows that the recommendations and alerts generated, derived from the application of the concurrent expert systems and their associated rules, do not have to match the expert’s opinion. The meaning of this would not be that the system is invalid but, on the contrary, that the system allows to amplify the discrepancy in the judgement by combining a large dataset that humans would need much time to analyze. In front of this discrepancy, the expert should assess the reasons why the objective data produces such judgment and, beyond checking for potential mismatches either in the inference process or in the starting data, question the power of her own assessment. In the case of an agreement of both judgements, the system would gain in trust and worthiness. Both cases, discrepancy and agreement, lead the methodology to acquire and incorporate the corresponding reinforcements through the adaptation of its calculation models and the inference systems’ own rules. In this sense, it might be worthy to highlight the methodology design in itself as a guarantee of this aforementioned adaptability. Thus:

- All the stages, from the collection of information to the fuzzification of the input and output variables, can be modified without harming the operation of the inference systems, and, therefore, without affecting the methodology procedures.
- The use of concurrent inference systems allows the expert, who is in charge of managing the second inference system, to assess the accuracy of the measurements that were taken autonomously and loaded into the first system as input variables. The expert can thus detect measurement errors caused by malpractice, manufacturing defects, or the inappropriate usage of the medical instruments involved.
- The second concurrent system, based on the expert’s assessment, is implicitly conditioned by the measurements system. That is, if the expert validates the measurements obtained and categorizes them as reliable, then they will have a greater weight on the final decision associated to the assessment of the treatment, and this will indirectly condition their evaluations by minimizing their errors and subjectivity. Thus, if the system for example detects a combination of technical criteria that advises for a certain change of treatment, it will be the expert who must remarkably change their evaluations to allow the results of the application of the methodology to change substantially. This must be argued and justified by the expert giving appropriate reasons, thus, conditioning her work to be strict and detailed. It would happen similarly in the opposite case, when the Technical Risk values advise to continue with the current treatment, where the expert must correct these indications according to several different options.
- The presented methodology is to be used as an on-the-spot tool, meaning that it works as a decision support tool within the healthcare evaluation process of pressure ulcers. Therefore, it concerns to the health expert to decide whether the behavior of the inference system is appropriate and if it is consistent with the usual distribution of cases. If not, she is granted the choice of either modifying the prior evaluations in the second inference system, or making corrections in the first and second level models. These corrections are in themselves tools to allow for reducing the error in the results by adjusting the calculation models of the decision factor behavior, while at the same time getting those results closer to reality and narrowing down the uncertainty in the models’ definition. Similarly, the second level corrections allow to manage the transition zones between the exponential model, which manages Technical Risk values up to 50, and the logarithmic model, applied when these values are over 50. There is in fact a fictitious alteration in the merging zone of both models, and that is the reason why the expert is offered a tool to modify it, adjusting it to the observed data.

According to all that has been explained, it has been proved that the flexibility at the time of modelling the inference engines of the proposed methodology allows its adaptation to multiple environments, and to a variety of decision criteria and perspectives. There are different approaches in the literature to the treatment of wounds as already described in Section 1. However, none of them show the characteristics described and presented in the displayed methodology, which, in turn, introduces a glossary of new terms: Technical Risk, Expert Risk, Decision Factor, and Global Risk, which make it possible to measure the quality and the effectiveness of the treatment according to the patient’s health status, factors that become reliable indicators for the expert in themselves. Their reliability lays in that, while in their technical aspect they coalesce a large set of input data, in their expert aspect they consolidate the expert’s own evaluations. Therefore, the methodology presented in this article fulfils all those formal aspects related to the treatment of PU, improving them with the incorporation of new behavior terms and models. All that, as explained with examples in Section 3, makes it possible to meet the initially outlined objectives, directly related to the improvement of the patient’s health status, and indirectly to the reduction of mistakes, length, and associated costs in treatments, as has been shown in this work.

## 5. Conclusions

The proposed methodology is very useful for the health field for which it was developed, well beyond its grounding as a decision support system based on expert systems because, among other merits, it makes it possible:

- To make a helping system available to healthcare personnel who might not be experts in the treatment and/or healing of chronic wounds, PU in this case.
- To make a system available to enable the monitoring of the healing process of PU, allowing such process to be performed with full knowledge of all its previous details, regardless of who performed the treatment before. This also facilitates comparisons between the PU status at different times.
- The potential application of the monitoring of PU (generally used in patients having a reduced mobility) both to outpatient and hospital care, this one reserved only to bedridden patients.
- To reduce the mistakes in the diagnosis and treatment stages of PU patients by providing the experts with tools allowing to streamline diagnosis work, and also to consolidate and coalesce the information available.
- To lower the length of treatments by reducing the number of evaluation mistakes and supervising the monitoring process, while at the same time cutting down the costs associated with the use of the appropriate healthcare equipment.

However, it must be highlighted that the proposed methodology is still in its early prototype stage, driving the need to continue to improve it, aiming to optimize its performance. In this line, it should be noted that:

- As already mentioned, the methodology is to be used as an on-the-spot tool, meaning that it does not record its decisions beyond a specific application associated to a particular moment in time. With that, the behavior of the inference system is not affected by any corrections other than those allowed during its application, and that limits its usefulness. The incorporation of an associated database and the definition of adaptive algorithms that could modify the behavior of the system between measurements depending on a set of pre-defined rules, would substantially improve the applicability of the methodology.
- The nature itself of the expert systems as autonomous decision-making elements is conditioned by the definition and the influence of the expert throughout the whole process. Although this autonomous behavior might be considered desirable, the specific importance of the environment where it is to be applied compels the participation of an expert in the final decision making. The reliability of an autonomous system in the recommendation of a PU diagnosis and the evaluation of its treatment is unavoidably limited by the potential consequences of a mistake. Even if this situation must still evolve, in this case the use of expert systems, together with the presence of an expert for reviewing and managing them, is considered acceptable.

The proposed methodology must evolve consistently, and keep incorporating new tools to improve the performance of those already embedded. In this sense, the inclusion of convolutional neural networks (CNNs) in the treatment of ulcer images is an alternative already subject to exploration and implementation. Additionally, the improvement in the definition of membership functions through automatic generation systems and the use of fuzzy vague sets are considered, at this moment, promising lines of research to complement the work presented.

Likewise, structures similar to those presented in this article can be transferred to other health applications, such as the treatment of glaucoma, viral infections, the detection of skin issues, etc. The procedure would, in essence, stay the same, needing only to redefine its behavior rules and models. It will be the usage of the methodology and its performance test in intensive work environments what will ultimately dimension its true scope, transforming it into a basic tool for the treatment of PU, while promoting a more cross-wise and widespread use for it.

## Figures and Tables

**Table 1:** Design needs, requirements, and the corresponding restrictions.

Follow-up of the Monitoring and Assessment Stages within the Pressure Ulcers (PU) Treatment Process		
Design needs for the artifact	Technical requirements	Restrictions associated to the environment
It must include a device to register information about the patient’s health status, regardless of her location	Mobile device with wireless data connectivity, screen, camera, keyboard and data templates	Mobile data service available at the patient’s location
It must have the ability to capture images of the wounds		
It must have the ability to transmit the information collected, as well as to receive information and upload it to the information system		
It must have the ability to manage the information about all the visits of the healthcare team to the patient, recorded on a historian	Graphical user interface	Visibility and resolution conditions of the graphic elements
It must have the ability to detect if the wounds under control increase or decrease its size in the period between visits	Image processing and subsequent calculations derived from said processing	Image control and quality
It must provide a metric corresponding to the global patient’s status, associated to the evaluation of the medical treatment	Braden, or similar, scale	Homogeneity in the assessment of the scale score by different healthcare professionals
It must make available all the information stored in the system, as well as any other coming from the treatments applied to the patient after the healthcare team	Computer equipment and software application with healthcare templates	IT support
It must combine the expert evaluations with the rest of the process information in the system	Expert systems that combine data coming from devices and data from healthcare assessment	
It must allow the processing of the information collected by the devices together with the evaluations of the expert establishing, based on them, recommendations regarding the treatment that the patient is currently undergoing		

**Table 2:** Description of the operation of the inference system used to calculate the Technical Risk.

Point	Step	Description
1	Definition of the fuzzy sets and the membership functions See Figure 6-Step 1	Firstly, the different fuzzy sets and the respective membership functions for each input variable of the system are established [62,63,64,65]. These functions make it possible to determine the degree of membership of a certain value to a specific set in the range from zero to one. This is equivalent, respectively, to not belonging at all, or fully belonging to, the qualitative trait that represents the section of the membership function. Trapezoidal functions have been chosen for this, since they make it possible to maximize the degree of belonging to a set within a continuous range of values.
2	Fuzzification of input variables See Figure 6-Step 2	The fuzzification process of the input variables makes possible to determine the degree of membership (in the range from zero to one) of a specific value, associated with a certain input variable, within a certain fuzzy set.
3	Determination of the rules See Figure 6-Step 3	After the fuzzy sets and the membership functions have been determined, and the fuzzification process of the input variables has been carried out, then the rules governing the behavior of the system are established. These rules allow to perform the combination of the antecedents, that is of the input values, by using fuzzy operators of the ‘AND’ and ‘OR’ types. After this, combinations of the ‘IF’… ‘AND’/‘OR’… ‘THEN’ … types are carried out, allowing to connect the input values with the consequents, i.e., the outputs of the system.
4	Evaluation and application of the rules See Figure 6-Step 4	After performing the previous steps described in this table, it is possible to apply the fuzzy operators. There are different methods for their application [62]. The ‘AND’ operator will be used with the ‘intersection’ fuzzy operator and the ‘OR’ operator with the ‘union’ one, also equivalent to the ‘minimum’ and ‘maximum’ operators, respectively. When the ‘AND’ connector is used to connect two membership functions, the minimum of their membership degrees is returned, while in the case of the operator ‘OR’ the maximum one is returned.
5	Obtaining the output fuzzy set See Figure 6-Step 5	After evaluating and applying the rules, the consequent of these rules is determined by using the implication method [62]. The minimum is chosen in this case, which is translated into a truncation of the consequent’s membership function, that has been previously defined as a fuzzy set based on the value obtained from the fuzzy operation.
6	Aggregation of consequents See Figure 6-Step 6	After evaluating all the rules and obtaining their individual consequents, the global consequent (representative of the risk) is determined.
7	Defuzzification See Figure 6-Step 7	Finally, a numerical value for the risk level is obtained by applying the centroid method [62,66,67].
